# Microscopic image dataset of fungal spores for computer vision applications in *Tectona grandis* and other taxa

**DOI:** 10.1038/s41597-025-06264-2

**Published:** 2025-12-11

**Authors:** Syeda Munjiba Islam, Md. Shariar Hossain Sazzad, Md. Faysal Ahamed, Maaz Ahmed, Sadikul Islam Nayon, Toufiq Islam, Sabrina Saniat Ayon, Julfikar Haider, M. Abdullah-Al-Wadud, S. M. Riazul Islam

**Affiliations:** 1 Department of Forestry & Environmental Science, Shahjalal University of Science and Technology, Sylhet, 3114 Bangladesh; 2 Department of Electrical & Computer Engineering, Rajshahi University of Engineering & Technology, Rajshahi, 6204 Bangladesh; 3 Department of Engineering, Manchester Metropolitan University, Chester Street, Manchester, M1 5GD UK; 4 Department of Software Engineering, College of Computer and Information Sciences, King Saud University, Riyadh, 11543 Saudi Arabia; 5 School of Natural and Computing Sciences, University of Aberdeen, Aberdeen, AB24 3FX UK

**Keywords:** Fungal biology, Scientific data

## Abstract

Fungal diseases pose significant threats to forestry species such as *Tectona grandis* (teak), as well as other important forestry and agricultural crops, highlighting the need for early and accurate identification of fungal spores, which serve as primary agents of dissemination and infection. Although Artificial Intelligence (AI) has enabled automated spore recognition, its effectiveness depends on the availability of large, diverse, and well-annotated datasets. However, publicly available datasets targeting fungal taxa associated with commercially valuable timber species remain scarce. To address this gap, we introduce a microscopic image dataset of fungal spores isolated from symptomatic teak foliage, including *Olivea tectonae*, *Colletotrichum siamense*, and *Neopestalotiopsis* sp. The dataset was developed through systematic field sampling, direct microscopic observation, and axenic culturing, followed by high-resolution imaging and manual annotation by experts. This annotated dataset serves as a foundational resource for AI-assisted spore detection across both field-based and atmospheric surveillance workflows, supporting applications such as sample-based analysis, air-based monitoring, and real-time diagnostics. Its cross-species utility and future extensibility enhance its value for plant disease management.

## Background & Summary

Teak, or *Tectona grandis*, is one of the highly valued timber species globally and is known for its durability, weather resistance, and aesthetic appeal. Native to India, Myanmar, Lao PDR, and Thailand, natural teak forests cover approximately 30.215 million hectares, with Myanmar holding the largest share. In addition to its native forests, teak has become a widely favoured species for plantations. Since the mid-1800s, teak plantations have been established globally, currently spanning approximately 4.854 million hectares, 80% of which are located in Asia^1^. Given its remarkable properties, teak is in high demand worldwide for a variety of uses, including furniture, shipbuilding, decking for boats, flooring, cabinetry, and exterior construction, making it one of the most versatile and sought-after timbers^1,2^.

Teak, owing to its long rotation period, remains vulnerable to biotic stresses throughout its growth^3^, with fungal pathogens being the most widespread and destructive. A recent global checklist has recorded 152 fungal species associated with teak across 39 countries, including frequently reported pathogenic genera such as *Olivea*, *Ganoderma*, *Fusarium*, *Cercospora*, *Colletotrichum*, and *Alternaria*^4^. These pathogens cause various diseases, including leaf rust, leaf spots, blight, and root rot, leading to premature defoliation, stunted growth, seedling mortality, and reduced timber quality^5,6^. Such infections are especially harmful in nurseries and young plantations, often resulting in significant productivity losses and presenting a persistent threat to successful teak cultivation.

This study compiles a microscopic image dataset of fungal spores derived from symptomatic teak leaves. The dataset encompasses spore images of *Olivea tectonae*, *Colletotrichum siamense*, and *Neopestalotiopsis* sp., and is intended to support the development of computational approaches for fungal detection and classification.

*Olivea tectonae*, the causal agent of teak rust, is a widespread and damaging pathogen affecting teak plantations across tropical and subtropical regions, including countries in South and Central America such as Panama, Costa Rica, Mexico, Colombia, and Brazil, Southeast Asia including India, Myanmar, Thailand, Laos, Cambodia, Vietnam and Bangladesh. The disease typically begins with small necrotic spots on the adaxial leaf surface, which enlarge and coalesce over time. Infected trees, especially young plantations, experience rapid and severe defoliation, often within weeks, leading to significant reductions in growth and timber yield. Across affected regions, disease incidence in plantations and nurseries has been reported to reach up to 100%, with severity levels frequently exceeding 70%, underscoring the global threat teak rust poses to sustainable teak cultivation^7–12^. Additionally, *Colletotrichum siamense* is linked to foliar infections in teak plantations and is recognized as a causative agent of diseases such as leaf spots, blight, and fruit rot in several other plant species, including *Dipterocarpus turbinatus*, *Theobroma cacao*, *Malus domestica*, and Alo vera^5,6,13–17^. *Neopestalotiopsis* sp. is also increasingly being reported as a fungal pathogen responsible for leaf spots, root rot, crown rot, and fruit rot across diverse plant species^18–22^.

The presence of these fungi can be identified through the detection of urediniospores produced by *Olivea tectonae* and asexual conidial spores produced by *Colletotrichum siamense* and *Neopestalotiopsis* sp. These spores not only serve as key diagnostic markers but also play a central role in fungal dissemination, commonly through air and water. Screening for these fungal spores in the atmosphere offers a valuable opportunity for early disease prediction and intervention. Under natural conditions, these fungal taxa are also known to form spore-bearing structures on infected plant surfaces^17,22,23^. In such cases, microscopic examination of field-collected samples can further support disease assessment. Traditionally, identifying fungal species based on spore morphology relies on visual inspection and manual counting. While this method is straightforward, it is time-consuming, labor-intensive, and prone to subjectivity^24^. With advancements in artificial intelligence, computer-aided technologies are now being employed to automate spore detection for early diagnosis. However, the effective implementation of these technologies depends on the availability of large and diverse datasets of fungal spores. In response, several research initiatives are now focused on constructing comprehensive image datasets of fungal spores and developing detection and classification techniques, which are being applied both in sample-based microscopic assessments and in real-time atmospheric surveillance under field conditions^25^.

For example, airborne spores of *Puccinia striiformis* were collected in a laboratory-simulated wheat field environment using portable volumetric spore traps and were then subjected to microscopic imaging. An algorithm incorporating K-means clustering, contour segmentation, and ellipse fitting was proposed for the automatic detection and counting of the spores, achieving an average accuracy of 98.6%^26^. Likewise, Tahir *et al*.^27^ developed an optical sensor system designed to capture images of airborne fungal spores under three different lighting conditions: bright-field, dark-field, and autofluorescence. To classify these spores, they applied handcrafted features in conjunction with Histogram of Oriented Gradients (HOG), utilizing a cubic-kernel SVM. Consequently, the system attained an accuracy of up to 88%, along with a 70% AUC on the precision-recall curve. Subsequently, to tackle the challenge of early detection of airborne fungal spores, a dataset of 40,800 images was created using an optical sensor system. These spore images, collected from the air via an air sampling unit, included five types of fungal spores as well as dirt, captured under varying lighting conditions. A convolutional neural network (CNN) trained on this dataset achieved an accuracy of 94.8% through five-fold validation, demonstrating its potential for precise and timely identification of fungal contamination^28^. Additionally, a microscopic image dataset of *Lasiodiplodia* species responsible for grapevine trunk diseases was constructed. The species were cultured to induce spore formation, and images of the spores were then captured under a microscope, resulting in a total of 1320 images (660 RGB and 660 grayscale). Deep learning models such as VGG-16, InceptionV3, MobileNet, and ResNet-50 were used for fungal species classification, aiming to enhance the early detection and management of grapevine trunk diseases^29^. To further advance fungal detection in agricultural settings, a dataset comprising microscopic images of powdery mildew, gray mold, and fusarium wilt spores was collected from infected cucumber leaves. The detection method utilized GCS-YOLOv8, an improved deep-learning model designed to accurately identify these spores despite challenges like complex backgrounds and varying lighting conditions. This method achieved an accuracy of 92.6%, supporting disease monitoring efforts^30^. Similarly, a study by Zhang *et al*.^31^ developed a dataset of 20,000 microscopic images of *Fusarium asiaticum* and *Fusarium graminearum*, sourced from indoor cultures and simulated field conditions. The images capture variations in spore shape, impurities, and environmental factors to enhance detection accuracy in practical scenarios. The researchers applied GSD-YOLO, an improved version of YOLOv7-Tiny featuring GSConv modules and a decoupled head, which delivered high accuracy and real-time performance in distinguishing between the two species supporting more effective wheat disease monitoring. Furthermore, a benchmark dataset was constructed to identify *Magnaporthe oryzae* spores responsible for rice blast disease and differentiate them from other microscopic fungal spores and impurities. The dataset consisted of 8,959 microscopic images of individual spore classes and 1,450 microscopic images of mixed spore classes, including rice blast spores and common impurities, collected from both indoor cultures and field conditions. Among the tested models for detecting and classifying fungal spores, YOLOv3_DarkNet53 demonstrated strong reliability in distinguishing *M. oryzae* spores despite environmental challenges^32^. Table 1 shows a comparison of recent studies on fungal spore detection and classification, highlighting their objectives, dataset characteristics, availability, and applied classification techniques.

**Table 1:** Compiled datasets for fungal spore detection and classification.

Reference	Objective	Class composition of the dataset	Dataset details	Availability	Classification techniques
Lei *et al*.^26^	Automatically detect and count urediniospores of *P. striiformis*	Single class (Urediniospores of *P. striiformis*)	150 microscopic images	Private	K-means, shape analysis, ellipse fitting
Tahir *et al*.^27^	Detect airborne fungal spores	Multiple classes (Various airborne fungal spores)	1928 RGB images via optical sensor system	Private	HOG and handcrafted features with SVM (cubic)
Tahir *et al*.^28^	Early detection of fungus	Multiple classes (5 spore types + dirt)	40,800 RGB images	Private	CNN (11-layer custom architecture)
Crespo-Michel *et al*.^29^	Fungal spore classification in grapevine	Four distinct classes (*L. brasiliensis*, *L. crassispora*, *L. exigua*, *L. gilanensis*)	1320 microscopic images	Private	ResNet-50, VGG-16, MobileNet, InceptionV3
Zhu *et al*.^30^	Detect cucumber pathogen spores in natural scenes	Three distinct classes (Powdery mildew, Gray mold, Fusarium wilt spores)	484 microscopic images	Public	GCS-YOLOv8
Zhang *et al*.^31^	Classify *Fusarium* spores causing wheat scab	Two distinct classes (*F. asiaticum*, *F. graminearum*)	20,000 microscopic images	Private	GSD-YOLO (Optimized YOLOv7-Tiny)
Zhou *et al*.^32^	Identify *M. oryzae* spores and differentiate them from impurities	Five distinct classes (*M. oryzae*, *Fusarium spp*., *Alternaria spp*., *Botrytis spp*., Rice Pollen)	8,959 microscopic images of individual spore classes and 1,450 microscopic images of mixed spore classes	Private	YOLOv3_DarkNet53

Despite considerable advances in computer-aided technologies for fungal spore detection and classification, there remains a significant lack of publicly available datasets focused on fungal taxa associated with commercially valuable timber species. To help bridge this gap, the present dataset was created, comprising microscopic spore images of three fungal species associated with teak. It is intended to support the integration of such technologies into fungal surveillance workflows, including sample-based and atmospheric assessments, with applications in teak plantation health monitoring and beyond. This article establishes a foundation for the systematic exploration of the dataset, detailing its construction, validation, and potential applications. Section 2 provides a detailed description of the methodology employed in constructing the dataset. Section 3 outlines the structure and hosting details of the dataset, ensuring accessibility for future use. Section 4 presents the technical validation procedures implemented to confirm the dataset’s consistency and reliability. Finally, Section 5 explores the potential research applications of the dataset, particularly in the context of teak plantation health and broader forestry management.

## Methods

### Data collection

#### Sample acquisition and microscopic imaging

**Fig. 1:**
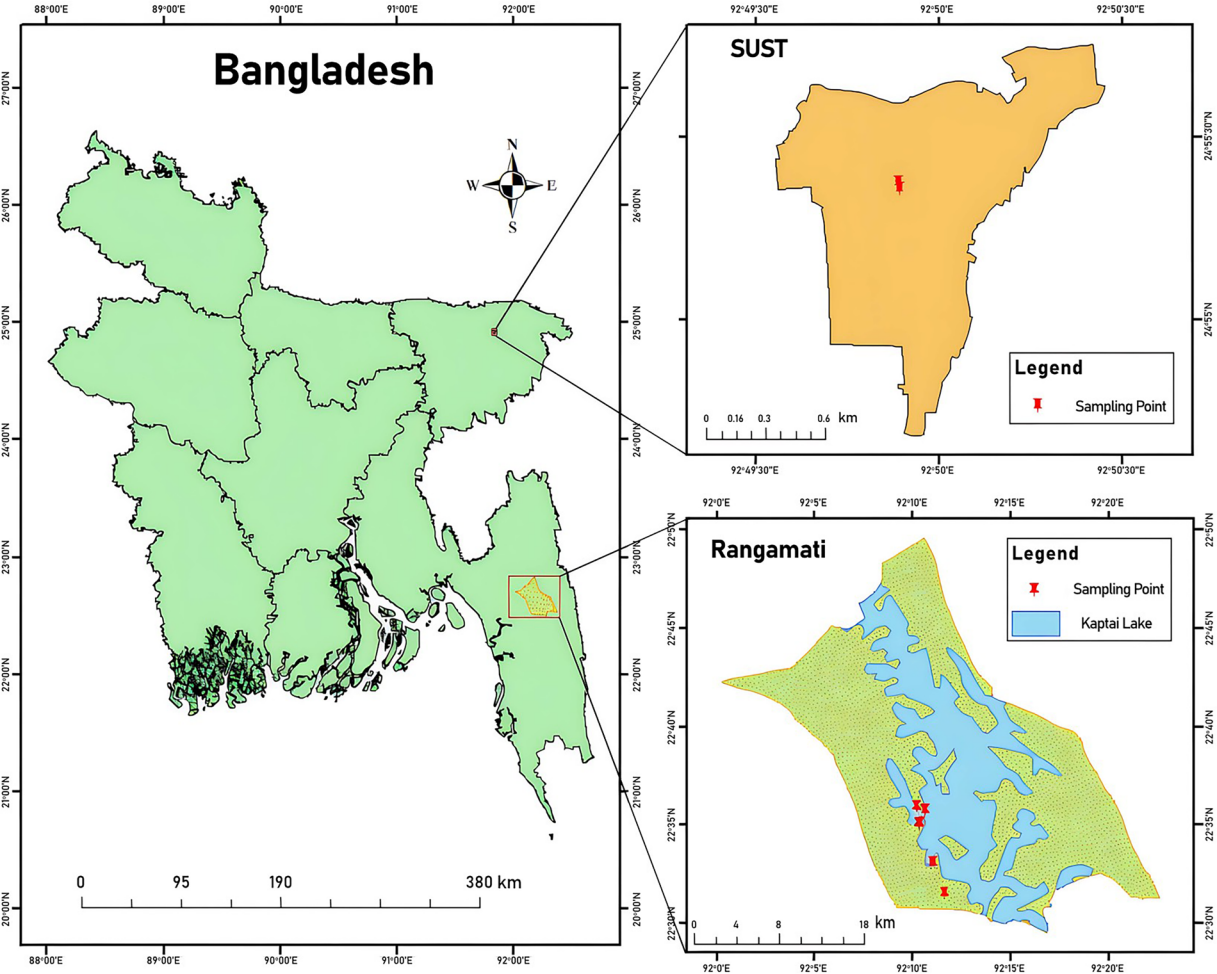
Geographical location of sampling sites for *Tectona grandis* leaf collection in Bangladesh. The main map shows the administrative boundaries of Bangladesh with highlighted regions indicating the two study locations: Shahjalal University of Science and Technology (SUST), Sylhet (top right), and Rangamati Forest Division, Kaptai (bottom right). Red pins represent specific sampling points where symptomatic teak leaves were collected.

Leaf samples of *Tectona grandis* were collected during the dry winter season (October–March) from two locations in Bangladesh: Shahjalal University of Science and Technology (SUST), Sylhet (24°55′21″N, 91°49′52″E), and Kaptai, Rangamati (22°38′N, 92°11′E) (Fig. 1). At each location, symptomatic teak leaves showing varying degrees of disease expression were collected from multiple trees. Samples were stored in Ziplock bags containing silica gel to preserve integrity during transport and storage prior to laboratory analysis.

**Fig. 2:**
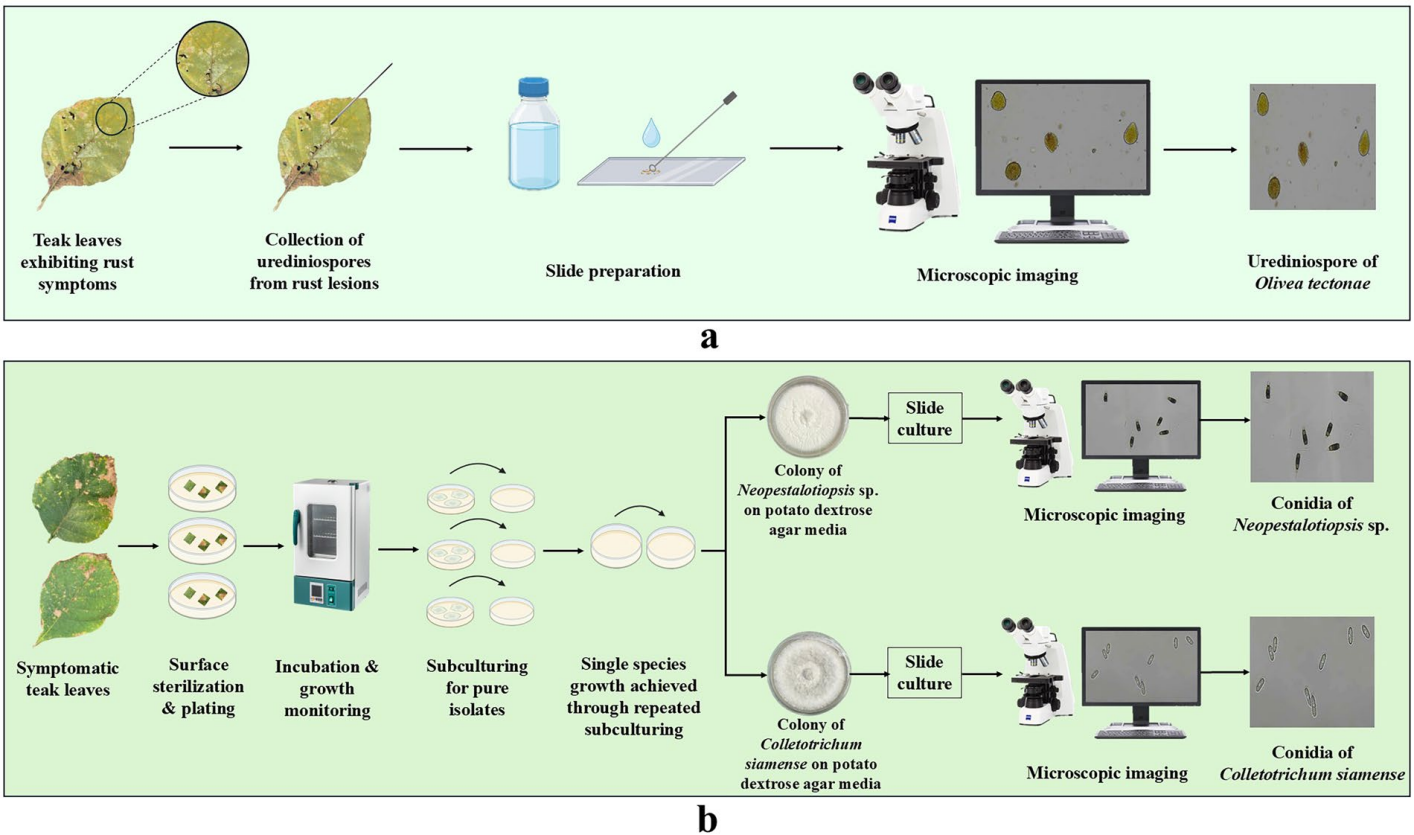
Flow diagram of sample acquisition and microscopic imaging. (**a**) Microscopic imaging of urediniospores of *Olivea tectonae* from rust-infected teak leaves. (**b**) Isolation, culture, and imaging of fungal spores from symptomatic teak leaves.

Among the collected leaf samples, some exhibited symptoms of rust caused by *Olivea tectonae*, characterized by gray necrotic spots on the adaxial leaf surface that gradually enlarged and coalesced into larger lesions. Numerous subepidermal uredinia were observed on the abaxial surface of these lesions, indicating the presence of urediniospores. To examine and document the urediniospores, they were carefully scraped from the infected regions on the abaxial surfaces of symptomatic teak leaves using a sterile inoculation loop. The collected spores were transferred onto clean microscope slides with a drop of sterile distilled water, and coverslips were gently applied to prepare wet mounts for microscopic observation. The slides were examined under a Zeiss Primostar 3 microscope at 40 × magnification to assess spore morphology and arrangement. High-resolution images were captured using a digital imaging system connected to the microscope via computer software, enabling detailed analysis and documentation. (Fig. 2a).

Other collected samples displayed distinct symptoms of leaf blight and leaf spot. Blight symptoms were characterized by expanding brown to grayish-brown necrotic lesions, primarily initiating near leaf tips and progressing along the margins, often leading to tissue collapse. Leaf spot symptoms, in contrast, appeared as discrete circular to irregularly shaped lesions scattered across the lamina. To ensure thorough documentation, symptomatic tissues representing both blight and spot types were processed separately. Leaf sections (~5 mm²) were aseptically excised from advancing lesion margins and surface-sterilized by immersion in 70% ethanol for 1 minute, followed by three rinses in sterile distilled water. The samples were then dried with sterile filter paper and plated on Potato Dextrose Agar (PDA). Each sample set (blight and spot) was plated in biological replicates.

The inoculated PDA plates were incubated at 25 ± 1 °C for 3–4 days, with observations conducted every 24 hours to monitor emerging fungal growth. By day 4, distinct colonies with varying morphological features had developed. Individual fungal colonies were aseptically subcultured onto fresh PDA plates and incubated under the same conditions. Successive subculturing over 7–10 days was carried out to ensure pure, axenic cultures were obtained, eliminating contaminants and mixed populations. Two such pure isolates were obtained and later identified through molecular characterization as *Neopestalotiopsis* sp. and *Colletotrichum siamense*, with identification details provided in a subsequent section.

**Fig. 3:**
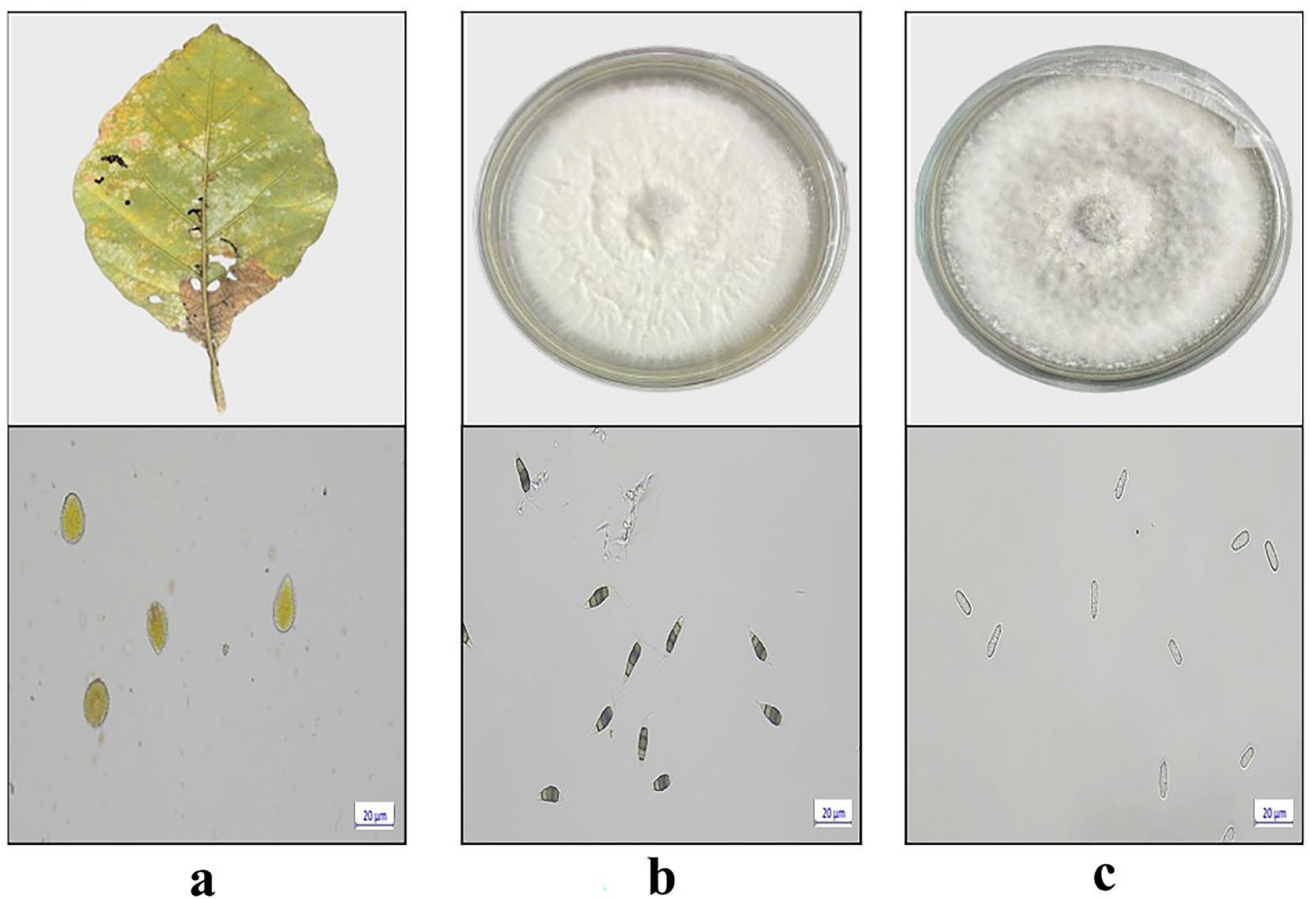
Representative images of rust-affected teak leaves, fungal colonies on potato dextrose agar (PDA), and corresponding spore morphologies observed under a microscope. (**a**) Teak leaf showing rust symptoms caused by *Olivea tectonae* and corresponding urediniospores (bottom) under a microscope. (**b**) Colony of *Neopestalotiopsis* sp. on potato dextrose agar media and conidia under a microscope (bottom). (**c**) Colony of *Colletotrichum siamense* on potato dextrose agar media and conidia under a microscope (bottom).

For the pure fungal isolates, spore imaging was performed using the slide culture method. A PDA block (~0.5 cm²) inoculated with each fungal isolate was placed on a sterile microscope slide, covered with a sterile coverslip, and maintained within a moist chamber setup. The chamber was prepared by lining a Petri dish with moistened sterile filter paper and supporting the slide on a U-shaped sterile glass rod to ensure aerial growth. These setups were incubated at 25 °C for 7–14 days, allowing sufficient time for sporulation. Coverslips were then carefully removed and placed on new slides with a drop of sterile distilled water to prepare wet mounts. The preparations were examined using a Zeiss Primostar 3 microscope at 40 × magnification, and high-resolution images of the spores were captured using a digital imaging system connected to the microscope. These images were systematically catalogued and added to the spore image dataset for documentation (Fig. 2b). Furthermore, to obtain images for evaluation as part of the test set, a mixed spore suspension was prepared to include combinations of different spore types. Urediniospores of *O. tectonae* were collected from mature uredinia on infected teak leaves and suspended in sterile distilled water. Conidia of *Neopestalotiopsis* sp. and *C. siamense* were collected from actively sporulating PDA cultures, suspended in sterile distilled water containing 0.01% Tween-20, and filtered to remove mycelial fragments. Equal volumes of the urediniospores and conidial suspensions were combined, and images of the mixed spores were captured using the same microscopic setup described above. All the required processes were performed in a laminar air flow chamber to maintain aseptic conditions. No staining was performed for the fungal isolates to adequately represent the field conditions and maintain their natural morphology. Figure 3 presents representative images of rust-affected teak leaves, cultured fungal colonies, and their corresponding spore morphologies.

### Molecular identification

**Fig. 4:**
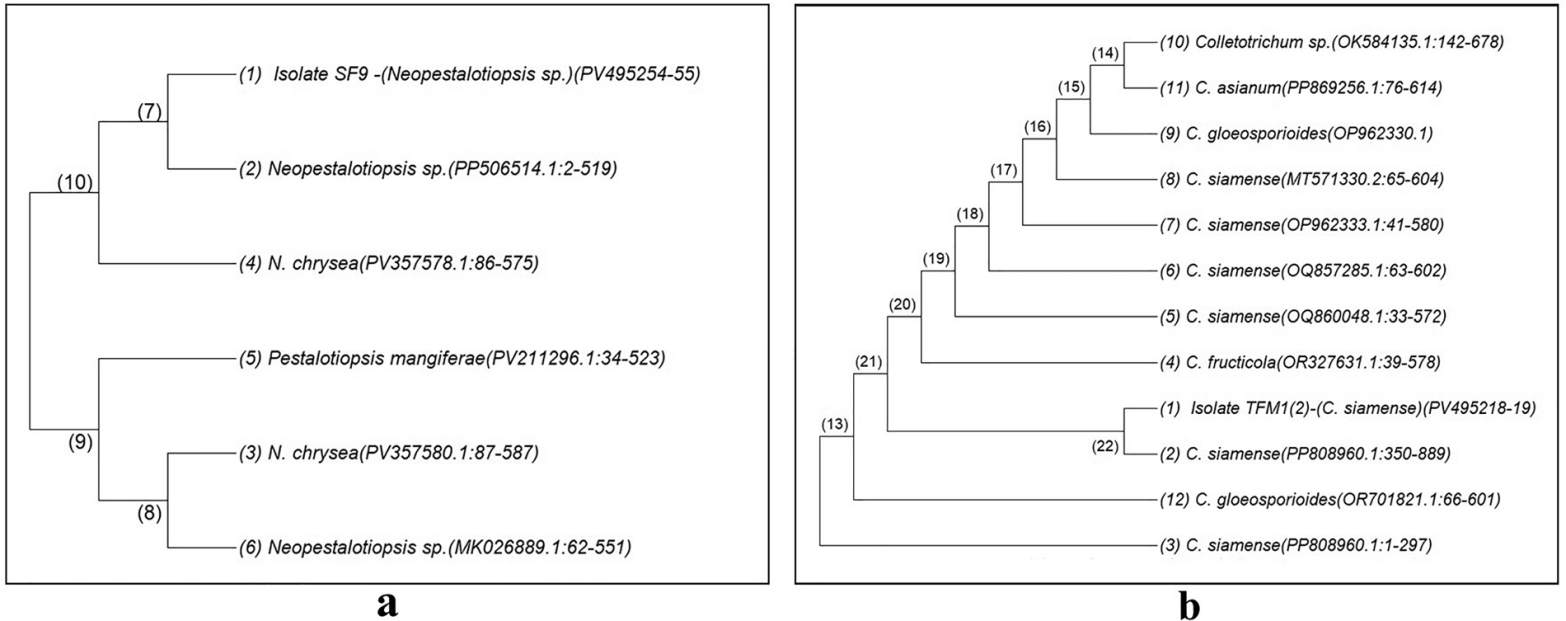
Phylogenetic relationships of fungal isolates SF9 and TFM1(2) with related taxa, based on internal transcribed spacer (ITS) region sequences. (**a**) Phylogenetic relationship of isolate SF9 with related taxa inferred using the Neighbor-Joining method. Each branch represents a fungal species or isolate, with numbered nodes (7, 8, 9, 10) indicating common ancestors. Closely related sequences are clustered together, and shorter branch lengths represent higher sequence similarity. The tree shows the position of isolate SF9 within the Neopestalotiopsis clade. (**b**) Phylogenetic relationship of isolate TFM1(2) with related taxa inferred using the Neighbor-Joining method. Each branch represents a fungal species or isolate, with numbered nodes (13 to 22) indicating common ancestors. Closely related sequences are clustered together, with shorter branch lengths representing higher sequence similarity. The tree shows the position of isolate TFM1(2) within the *Colletotrichum siamense* species complex.

For molecular identification, conserved regions, namely, internal transcribed spacer 1 (ITS1), 5.8S ribosomal DNA, and ITS4 were sequenced from the genome of each isolate using the universal primers ITS1 (forward) and ITS4 (reverse). The obtained sequences were assessed using BLASTn (https://blast.ncbi.nlm.nih.gov/) to determine species identity. The analysis revealed that the sequence of Isolate 1 (labeled Sample-SF-9; GenBank Accession No. PV495254–55) was 100% identical to that of *Neopestalotiopsis* sp. (GenBank Accession No. PP506514), and the sequence of Isolate 2 (labeled Sample-TFM1(2); GenBank Accession No. PV495218–19) was 100% identical to that of *Colletotrichum siamense* (GenBank Accession No. PP808960). Related sequences were retrieved from GenBank for phylogenetic analysis, and evolutionary trees were constructed for the isolates (Fig. 4) using MEGA 12 with ClustalW alignment under default parameters. The sequences of the isolates were deposited in GenBank (https://www.ncbi.nlm.nih.gov/nucleotide/) under the accession numbers PV495254–55 and PV495218–19.

### Dataset description

**Fig. 5:**
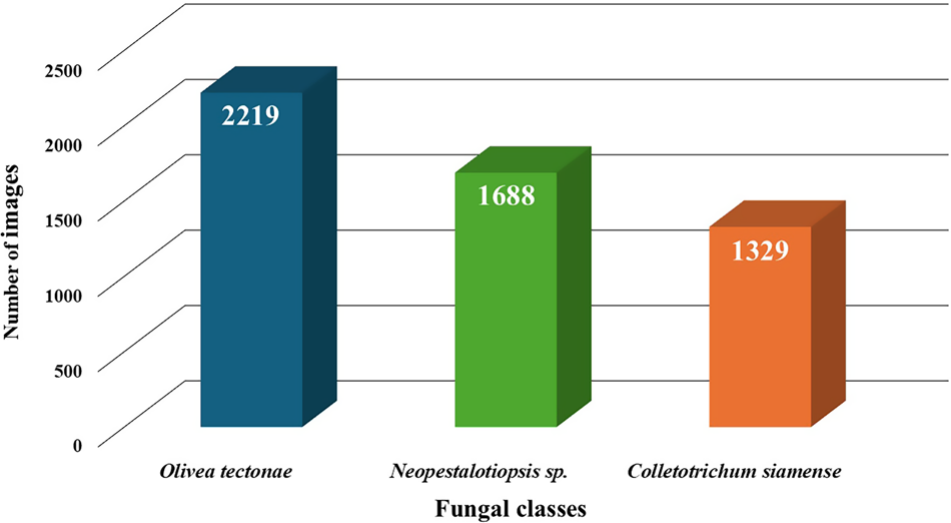
Distribution of total images per class. (**a**) Urediniospores of *Olivea tectonae*, represented by the blue bar. (**b**) Conidia of *Neopestalotiopsis* sp., represented by the green bar. (**c**) Conidia of *Colletotrichum siamense*, represented by the orange bar.

The dataset^33^, designated as the “*Tectona grandis* Fungal Community (TgFC)”, comprises 5,236 microscopic images of fungal spores, each standardized to a resolution of 640 × 640 pixels. These images represent three fungal taxa: *Olivea tectonae*, *Neopestalotiopsis* sp., and *Colletotrichum siamense*. As shown in Fig. 5, the dataset includes 2,219 images of urediniospores of *O. tectonae*, and 1,688 and 1,329 images of conidial spores of *Neopestalotiopsis* sp. and *C. siamense*, respectively. In addition, a separate set of 30 images containing mixed-class spores was curated and reserved exclusively for testing purposes, allowing evaluation of model performance in complex real-world scenarios where multiple fungal taxa may co-occur in the same field of view. The dataset consists of raw, unprocessed images of fungal spores. No preprocessing steps, such as normalization, contrast adjustment, or augmentation, have been applied. Researchers may apply any image processing or analytical techniques as required for their specific workflows.

### Data annotation

**Fig. 6:**
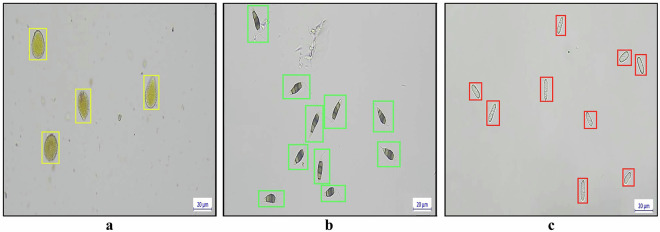
Annotation of fungal spores for object detection. (**a**) Urediniospores of *Olivea tectonae*, annotated with yellow bounding boxes. (**b**) Conidia of *Neopestalotiopsis* sp., annotated with green bounding boxes. (**c**) Conidia of *Colletotrichum siamense* annotated with red bounding boxes.

Fungal spores were annotated for object detection using LabelImg, a commonly used image annotation tool. Each spore instance was manually annotated using rectangular bounding boxes, which tightly enclose individual spores while minimizing background content. Most spores, including partially visible structures, were annotated consistently, and only extremely unclear or unidentifiable spores were excluded. This approach ensures comprehensive coverage of the dataset while maintaining high annotation quality. The annotation of spore classes was based on the distinct morphological and pigmentation characteristics of each species. Urediniospores of *Olivea tectonae* were identified by their round to elliptical shape and orange-yellow coloration, which is characteristic of this species (Fig. 6a). Conidia of *Neopestalotiopsis* sp. were recognized by their fusiform to cylindrical shape, typically comprising five cells: two hyaline end cells and three versicolorous median cells, along with apical setulae (hair-like appendages), distinguishing them from other spore types (Fig. 6b). Conidia of *Colletotrichum siamense* were characterized by their hyaline, aseptate, smooth-walled, cylindrical shape with rounded ends (Fig. 6c). These morphological distinctions facilitated precise spore labeling and classification, improving the dataset’s suitability for computer vision object detection.

## Data Records

The dataset^33^ is hosted on Figshare (10.6084/m9.figshare.28855910) under CC BY 4.0 license and is organized in a single compressed directory structured for object detection tasks. The folder contains three standard data splits, training, validation, and testing, each of which includes two subdirectories: images for the input images and labels for the corresponding YOLO-format annotation files. An 80%-10%-10% split was applied for training, validation, and testing, respectively. Additionally, a separate folder containing 30 mixed-class images has been included. These images feature co-occurring spores from multiple fungal taxa within a single field of view and are reserved for testing purposes, providing a more complex scenario for model evaluation. The distribution of image counts for each class across these subsets is presented in Table 2.

**Table 2:** Distribution of images in the dataset across training, validation, and testing subsets for each fungal class.

Class	Total Images	Training (80%)	Validation (10%)	Testing (10%)
*Olivea tectonae*	2219	1787	224	208
*Neopestalotiopsis* sp.	1688	1350	169	169
*Colletotrichum siamense*	1329	1077	119	133
**Total**	**5236**	**4214**	**512**	**510**

## Technical Validation

Technical validation was carried out to ensure the dataset’s credibility for use in detection algorithms. Although specifically validated using YOLO models, the results confirm its applicability to a wide range of other detection algorithms. Nine YOLO models (YOLOv5s/m/l, YOLOv8s/m/l, YOLO11s/m/l) were trained using an 80%-10%-10% split for training, validation, and testing. Training was conducted for 100 epochs with a batch size of 32 and a constant learning rate of 0.01, incorporating early stopping (patience 10) to prevent overfitting. A composite loss combining bounding box, class, and object detection errors guided the training, enabling reliable automated detection of fungal spores.

### Evaluation metrics

Model performance was evaluated using Precision, Recall, F1-Score, and mean Average Precision (mAP_50_). A detection is counted as a true positive if the Intersection over Union (IoU) with ground truth is ≥0.5. Precision, Recall, and F1-Score are defined as:

1 $${Precision}=\frac{{TP}}{{TP}+{FP}}$$

2 $${Recall}=\frac{{TP}}{{TP}+{FN}}$$

3 $$F1{Score}=2\ast \frac{{Precision}\ast {Recall}}{{Precision}+{Recall}}$$

F1-Score balances false positives and false negatives. mAP_50_ summarizes overall precision–recall performance by averaging the Average Precision (AP) of all classes. AP is computed as the area under the precision–recall curve using 11 fixed recall points:

4 $$A{P}_{11}=\frac{1}{11}{\sum }_{R\in [0,0.1,\ldots ,0.9,1]}{P}_{interp}(R)$$

5 $${mAP}=\frac{1}{N}{\sum }_{i=1}^{N}A{P}_{i}$$

Where N is the number of classes and AP_i_ is the average precision for the i-th class, which is calculated from the area under the corresponding precision-recall curve.

### Performance evaluation

Table 3 presents the performance metrics of nine YOLO models trained on the dataset, including precision, recall, F1 score, and mean Average Precision at IoU 0.5 (mAP_50_). These metrics provide reference values demonstrating that the dataset contains sufficient information to train and evaluate object detection algorithms. Larger network architectures, such as YOLOv5*l* and YOLO11*l*, achieved higher metric values.

**Table 3:** Performance of the YOLO models.

Models	Precision	Recall	F1 score	mAP_50_
YOLOv5*s*	0.881	0.945	0.912	0.899
YOLOv5*m*	0.867	0.949	0.906	0.907
YOLOv5*l*	0.882	0.949	0.914	0.903
YOLOv8*s*	0.874	0.928	0.90	0.893
YOLOv8*m*	0.88	0.928	0.903	0.895
YOLOv8*l*	0.868	0.943	0.904	0.90
YOLO11*s*	0.883	0.944	0.913	0.896
YOLO11*m*	0.865	0.956	0.908	0.909
YOLO11*l*	0.875	0.959	0.915	0.907

**Fig. 7:**
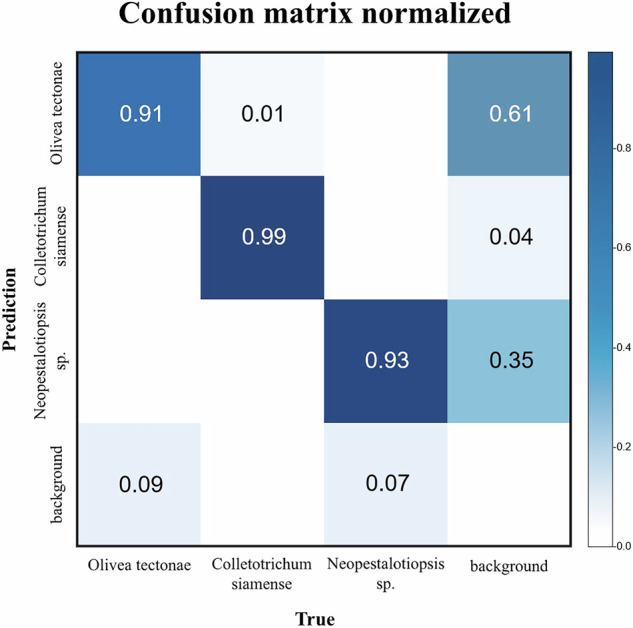
Normalized confusion matrix of YOLO11*l*.

Figure 7 shows the normalized confusion matrix of the YOLO11*l* model, presenting the prediction distribution across all classes. *Olivea tectonae* was correctly classified in 91% of instances, with minor predictions as background (9%) and *Colletotrichum siamense* (1%). *Colletotrichum siamense* was correctly identified in 99% of instances. *Neopestalotiopsis* sp. achieved 93% correct classification, with 7% of instances predicted as background. However, the background class showed notable confusion, with 61% incorrectly predicted as *Olivea tectonae* and 35% as *Neopestalotiopsis* sp. YOLO detects only labeled classes; all other regions are treated as the background class, which represents any area not belonging to the labeled objects, potentially causing false positives or missed detections.

**Fig. 8:**
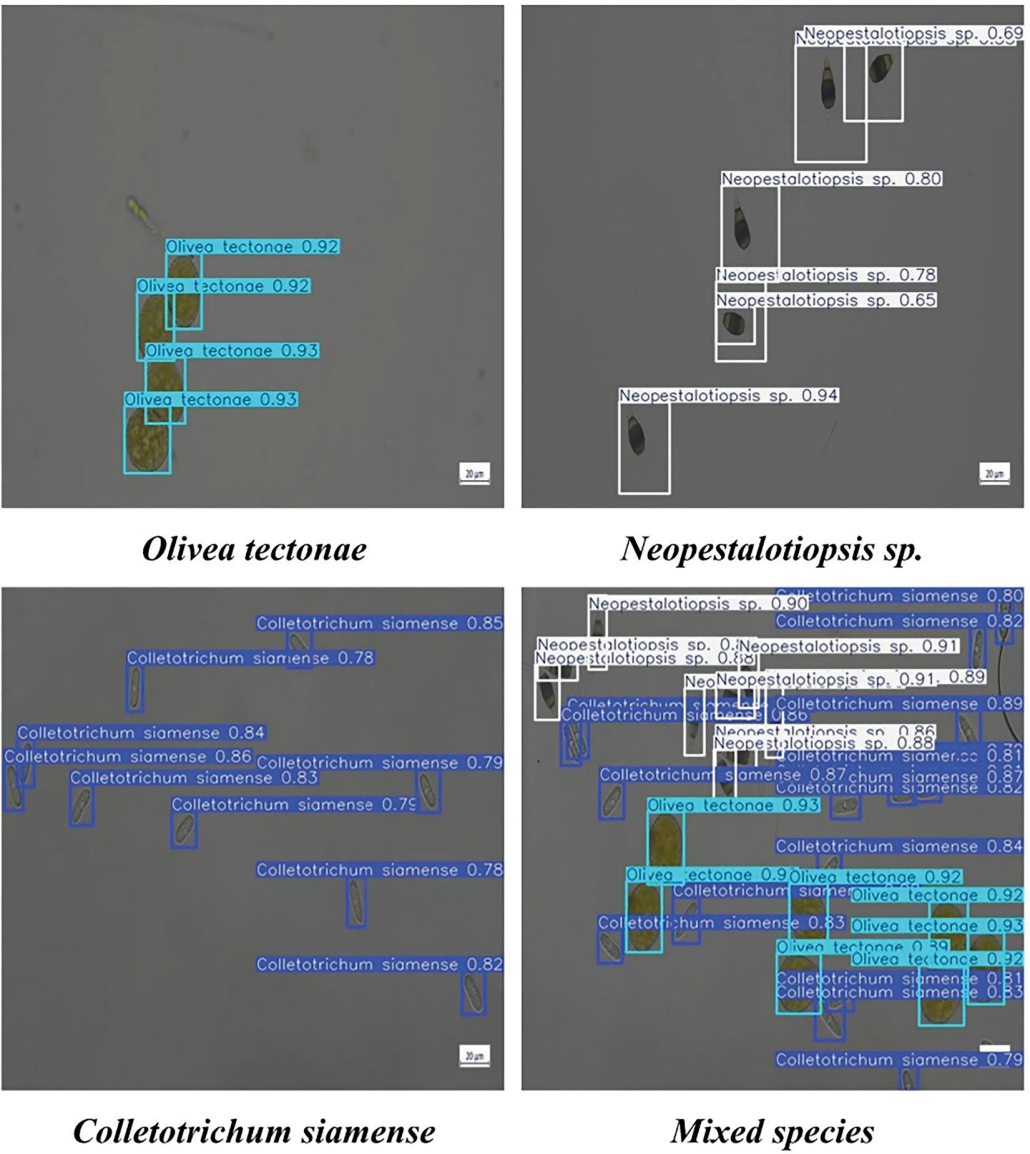
YOLO11l model performance. Predictions from YOLO11*l* model on class-specific images and an image of mixed spore classes.

Figure 8 shows the detection results from YOLO11*l* across three representative test images, each corresponding to one fungal spore class: *Olivea tectonae*, *Neopestalotiopsis* sp., and *Colletotrichum siamense*. For each class, predictions are visualized. YOLO11*l* produced high-confidence detections with accurate class assignments, with confidence scores above 90% in most cases. Additionally, a test image containing mixed spore classes is included, and the model’s detection results on this image showed accurate identification of multiple co-occurring spores with high confidence.

To assess the stability of dataset partitions, alternative train–validation–test splits of 70–20–10 and 60–30–10 were generated in addition to the original 80–10–10 split. Table 4 reports the model outputs on these splits, showing values for precision, recall, F1 score, and mean Average Precision at an IoU of 0.5. The consistency of precision, recall, F1-score, and mAP_50_ across these configurations indicates balanced class distributions and minimal partition-induced bias, supporting the dataset’s reliability and generalizability.

**Table 4:** YOLO11*l* model performance on different train–validation–test splits of the TgFC dataset.

Model	Split Ratio (Train–Valid–Test)	Precision	Recall	F1-Score	mAP_50_
YOLO11*l*	80–10–10	0.875	0.959	0.915	0.907
	70–20–10	0.865	0.950	0.908	0.898
	60–30–10	0.850	0.940	0.895	0.885

### Model interpretability with EigenCAM

**Fig. 9:**
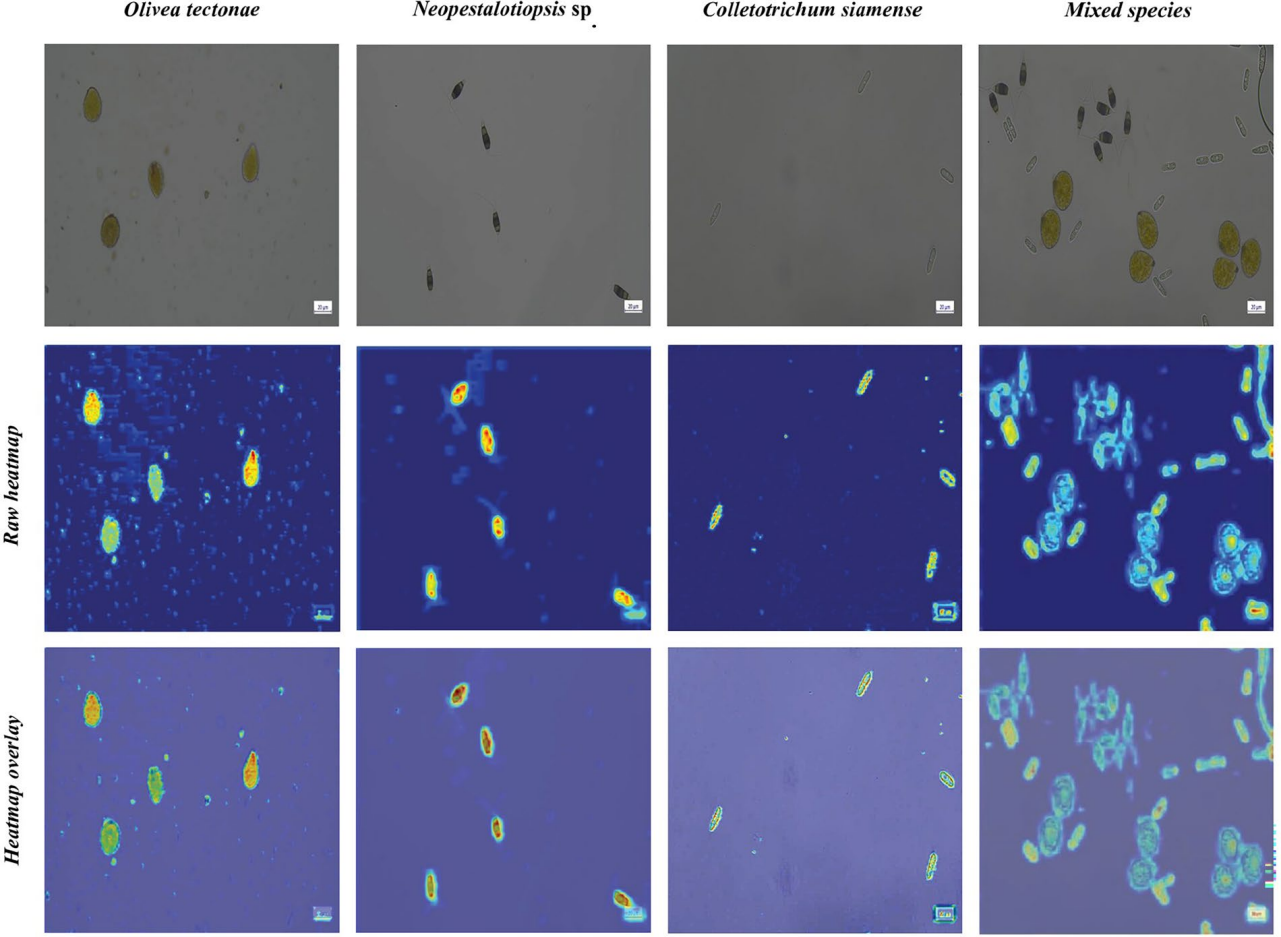
EigenCAM visualization. Class-specific and mixed class feature activations and heatmap overlays for YOLO11l model.

EigenCAM was employed to analyze the model’s focus areas during detection, which are visualized in Fig. 9. The heatmap generated using EigenCAM provides a visual explanation of the model’s decision-making process by highlighting regions of interest within the input data. EigenCAM uses principal component analysis (PCA) to decompose feature maps from the final convolutional layer into eigenvectors. These eigenvectors are then used to create class activation maps, which emphasize areas most relevant to the model’s predictions. The resulting heatmap employs a color gradient, where warmer colors (e.g., red) indicate greater relevance, and cooler colors (e.g., blue) signify lower relevance. These visualizations showed that the models primarily attended to distinctive spore morphology and spatial patterns, with YOLO11*l* producing comparatively concentrated activations around key structural features across different fungal groups.

### Preprocessing possibilities

**Fig. 10:**
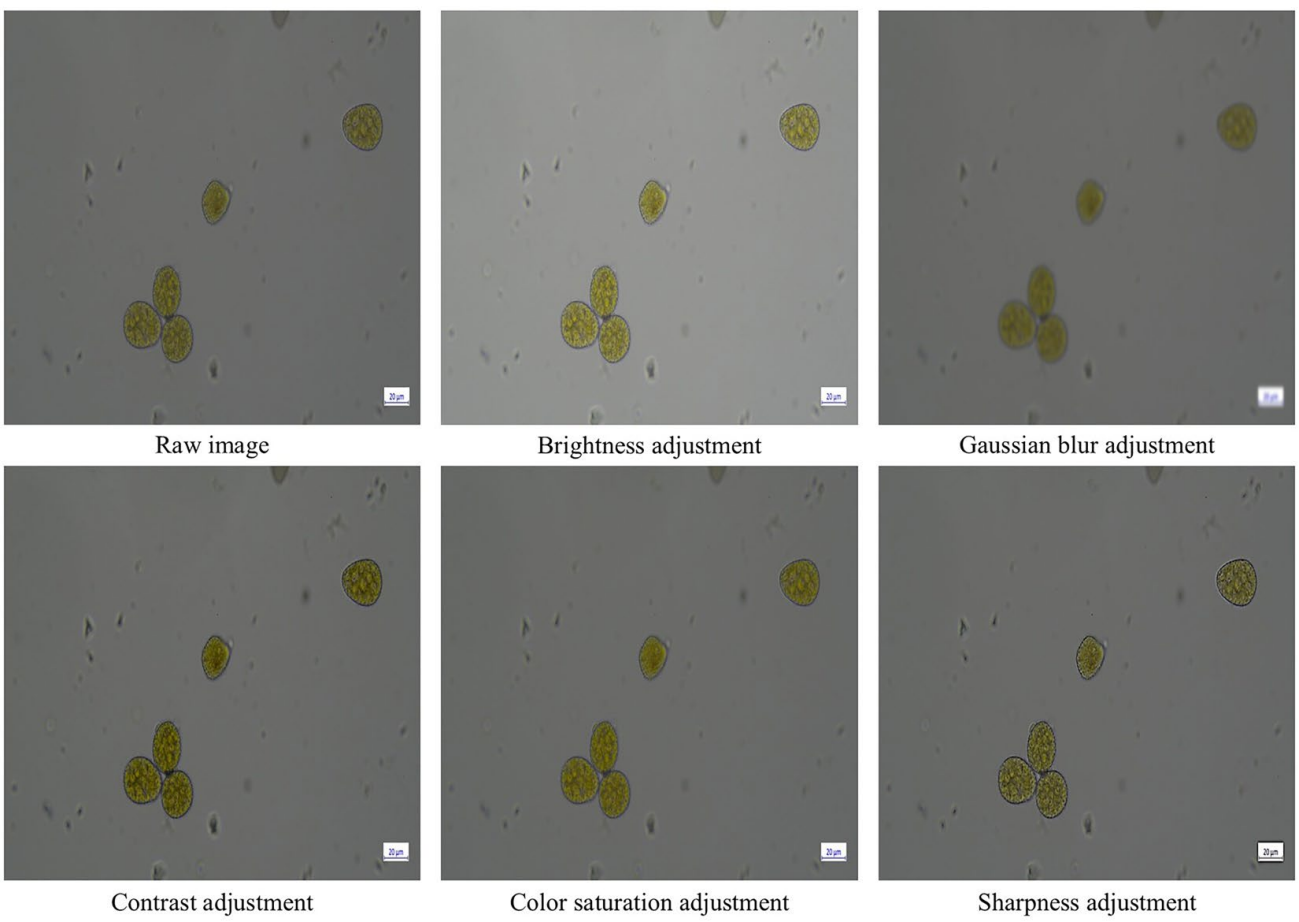
Examples of image augmentation techniques applied to the TgFC dataset.

Several preprocessing techniques can be applied to the TgFC dataset^33^ depending on the model architecture and research objectives. Pixel intensity can be standardized using image normalization, which can support training stability and model convergence. Contrast enhancement techniques can be applied to improve the visibility of key morphological features, such as spore borders, in response to variations in microscopic lighting. Image augmentation can also be applied to increase variation in the orientation and appearance of spores. Common augmentation approaches include random rotations, horizontal and vertical flips, scaling, adjustments in brightness and contrast, color saturation modifications, and Gaussian blur. Figure 10 presents examples of some of these preprocessing and augmentation techniques.

## Usage Notes

This dataset was designed to support AI-assisted fungal detection workflows in both field and atmospheric contexts. It serves as a versatile resource for researchers, educators, and developers working on plant disease surveillance, offering numerous practical and research-driven applications. The dataset is particularly relevant for *Tectona grandis* plantations, where early and accurate spore detection is crucial for disease management and yield protection. Beyond teak, its applicability extends to various agricultural and forestry ecosystems, supporting broader plant health monitoring and bioaerosol analysis across diverse environments.

### Automation of sample-based assessments

Under natural conditions, the fungal taxa encompassed within this dataset, *Olivea tectonae*, *Colletotrichum siamense*, and *Neopestalotiopsis* sp., are known to produce spore-bearing structures on infected foliar surfaces^9,22,23^. In such cases, foliar samples can be systematically collected and prepared for microscopic examination. The availability of a pre-annotated dataset enables the development of advanced object detection algorithms capable of automating the detection and identification of fungal spores directly from field-collected samples. This automation streamlines the process, providing rapid, reliable, and scalable identification of pathogens, thus overcoming the time constraints and inherent subjectivity associated with manual assessment.

### Air based sampling and classification

Airborne spore sampling and classification can be significantly enhanced through the use of a pre-labeled spore image dataset. Machine learning models pre-trained on this resource can detect and classify fungal spores collected from the air using systems like volumetric spore traps^26^ or air samplers^27,28^. Once trained, these models minimize the need for manual analysis, accelerating the detection workflow and enabling more efficient monitoring in plantation and forestry settings while supporting timely intervention.

### Integration into real-time surveillance systems

This dataset can be integrated into real-time systems for monitoring airborne spores of fungal pathogens around plantations, enabling prompt intervention. For example, systems like the Swisens Poleno device use digital holography combined with machine learning to capture high-resolution images of airborne particles, which can then be processed using a pre-trained classifier to identify fungal spores^34^. The access to an annotated dataset containing various fungal species enables the training of classifiers capable of detecting multiple fungal species in real-time. The dataset facilitates model training and real-time deployment, enabling faster response.

### Quantification methods

Object detection algorithms can be employed to not only automate the detection of fungal spores but also enable their precise counting. This automated approach significantly improves the accuracy and efficiency of spore counting compared to traditional manual methods. When spores are collected from the air using volumetric spore traps or similar devices, counting provides valuable insights into their concentration in the atmosphere^26^. The presence of an annotated dataset containing various fungal species supports the development of such algorithms capable of detecting and counting spores from multiple species. Monitoring spore concentrations is critical for early detection of potential fungal disease outbreaks, allowing for proactive management.

### Cross species utility beyond *Tectona grandis*

Although the dataset was created using samples from teak plantations, the fungal taxa it includes-*Colletotrichum siamense*^13–16^ and *Neopestalotiopsis* sp.^18–22,35^, are commonly found in a variety of agricultural and forestry settings. As a result, the dataset is widely applicable for developing general-purpose models to detect spores across different host plants, making it valuable for crop protection and forest health monitoring across various environments.

### Future extensibility

The open-access dataset is available via Figshare^33^, allowing researchers to download the images and, if desired, annotate and incorporate additional spore classes for use in their own research contexts. This flexibility enables the dataset to grow as a reference resource and accommodate a wider range of fungal taxa across diverse forestry and ecological settings. In addition, the authors plan to expand the dataset over time by incorporating new species and, in particular, additional mixed-culture images to enhance its coverage and representativeness.

### Training and benchmarking deep learning models

The dataset offers a foundation for training and benchmarking deep learning models, including convolutional neural networks (CNNs) and YOLO-based architectures, tailored for fungal spore detection. Its high-quality annotations and diverse spore morphologies allow researchers to build and validate reliable detection pipelines. Moreover, the dataset supports standardized evaluation, facilitating consistent comparison of model performance across different computational approaches.

### Model generalization and transfer learning

The dataset also holds potential for transfer learning applications. Models pre-trained on this dataset can be fine-tuned for detecting spores of other fungal taxa or adapted to new imaging environments, including variations in light source, microscope magnification, or camera resolution. This flexibility allows researchers to generalize existing models across broader taxonomic groups and environmental conditions, accelerating the development of spore detection systems for diverse plant-pathogen systems.

### Development of portable image-based diagnostic tools

The dataset serves as a foundational resource for portable, camera-assisted diagnostic applications. When paired with a microscope or macro lens, mobile devices could run lightweight models trained on this dataset to facilitate *in-situ* fungal diagnosis by field officers or technicians, even in low-resource settings.

## Data Availability

The dataset^33^ generated and analyzed in this study, comprising images of fungal spores along with YOLO-format annotation files for object detection, is publicly available on Figshare at 10.6084/m9.figshare.28855910.

## Code availability

All source code used for training, evaluation, and visualization is publicly available in a GitHub repository at https://github.com/MaazAhmed32/TgFC-Tectona-grandis-Fungal-Community-Dataset-Code-Validation and is released under the MIT license. The repository includes scripts for model training, result visualization using EigenCAM, and tools for dataset preparation and evaluation.
